# Single Prolonged Stress induces ATF6 alpha-dependent Endoplasmic reticulum stress and the apoptotic process in medial Frontal Cortex neurons

**DOI:** 10.1186/s12868-014-0115-5

**Published:** 2014-10-21

**Authors:** Bo Yu, Lili Wen, Bing Xiao, Fang Han, Yuxiu Shi

**Affiliations:** PTSD laboratory, Department of Histology and Embryology, Institute of Pathology and Pathophysiology, China Medical University, Shenyang, China; Department of Neurosurgery, Shengjing Hospital, China Medical University, Shenyang, China; Institute of pathology and pathophysiology, Department of Histology and Embryology, Basic Medical Sciences College, China Medical University, 92 North 2nd Road, Shenyang, 110001 Liaoning Province P.R. China

**Keywords:** Single-prolonged stress, Post-traumatic stress disorder, Medial prefrontal cortex, Activating transcription factor 6α, Glucose-regulated protein, ERP57, Caspase, Rat

## Abstract

**Background:**

In our previous researches, we have found that apoptosis was induced in the medial prefrontal cortex (mPFC) of post-traumatic stress disorder (PTSD) rats. Endoplasmic reticulum (ER) stress-induced apoptosis has been implicated in the development of several disorder diseases. The aim of this study was to investigate whether endoplasmic reticulum-related pathway is involved in single-prolonged stress (SPS) induced apoptosis in the mPFC of PTSD rats by examining the expression levels of ATF6 alpha (ATF6α), two important downstream molecular chaperones of ATF6α in the ER stress: Glucose-regulated protein (GRP) 78 and ERP57, and apoptotic factors caspase 12, caspase 9, and caspase 3.

**Results:**

Our results of Morris Water Maze (MWM) test showed that after SPS exposure, a striking increase of the escape latency was observed in SPS rats at day 1 through day 6, and SPS rats had much less time spent in target quadrant compared to control rats ( P < 0.01). And From immunofluorescence assays, we found that there was a gradual increase on the protein expression of ATF6α in response to SPS, which indicated ATF6α was activated by SPS. And additionally, immunohistochemistry assays, western blotting and reverse transcription-polymerase chain reaction (RT-PCR) showed that the immunoreactivity, protein and mRNA expression of GRP78 and ERP57 increased on 1, 4 days, and peaked on 7 days after SPS exposure, which revealed that SPS triggered inductions of GRP78 and ERP57 in the mPFC neurons. Moreover, RT-PCR assays demonstrated that there were up-regulations in the transcripts levels of caspase 12, caspase 9, and caspase 3 in response to SPS, which were according with the proteins changes of these apoptotic factors and indicated that ER stress and the activation of caspases contributed to SPS.

**Conclusion:**

Current data in this study highlight that SPS induced ATF6α-dependent Endoplasmic reticulum stress and ER-related apoptosis in the mPFC neurons, which indicated that the endoplasmic reticulum pathway may be involved in PTSD-induced apoptosis.

## Background

PTSD is an unique psychiatric disorder in that it is the result of a traumatic life event, and characterized by four clusters of symptoms: re-experiencing of fear memories, avoidance of trauma reminders, hyperarousal symptoms and negative alterations in cognition and mood [1-3], which may appear after a few days or months, even last several years [4,5]. Over the past several years, neuroimaging studies of PTSD have reported less activation or even deactivation in the medial prefrontal cortex (mPFC) regions during traumatic script-driven imagery in PTSD [6-8], and emerging studies showed that PTSD patients had a smaller mPFC [9]. And all these reports have been implicated that less activation of mPFC is associated with the pathogenesis of PTSD, and has played a key role in this disorder.

In the past several years, our research team have made great efforts on the dysfunction of the mPFC in the study of PTSD, and found there were apoptosis morphological changes by transmission electron microscopy (TEM) in the mPFC neurons of single prolonged stress (SPS)-model rats [10], which is a reliable animal model of PTSD, exhibiting behavioral abnormalities of PTSD and inducing enhanced inhibition of the hypothalamic–pituitary–adrenal (HPA) axis [11], as is a putative neuroendocrinological hallmark of PTSD [12]. However, the exact apoptosis mechanisms in the mPFC neurons remain poorly understood and require a deeper understanding of the cellular and molecular responses to SPS in order to reveal the pathophysiology changes of PTSD.

Apoptosis is also called programmed cell death. In recent years, endoplasmic reticulum stress (ERS) pathway is discovered as another important pathway of apoptosis [13]. ER is an essential intracellular organelle which is responsible for the synthesis and maturation of cell surface and secretion proteins, and maintenance of Ca^2+^ homeostasis. Disruption of these physiological functions leads to accumulation of unfolded proteins and induces ER stress. At the beginning, ER stress triggers the adaptative pathway, unfolding protein response (UPR) [14,15], which is essentially carried out by utilizing three types of ER stress sensor proteins, PERK (double stranded RNA-activated protein kinase-like ER kinase) [16], IRE1α (inositol requiring kinase 1α) [17], and ATF6α (activating transcription factor 6α) [18], and results in the upregulation of ER-derived chaperones and protein-folding enzymes leading the misfolded proteins undergoing the process of degeneration, such as an increase of glucose-related protein (GRP) family, such as GRP78/Bip, and the protein disulfide isomerase (PDI) family, such as ERP57/GRP58 [19,20].

Under normal conditions, ER chaperone GRP78 directly interacts with all three ER stress sensors, PERK, ATF6 and IRE1, and maintains them in inactive forms in non-stressed cells [21], and ERP57 plays a key role as folding catalysts [22]. And when ER stress occurs, GRP78 is released and allows the activation and transduction of the unfolded protein signals across the ER membrane to the cytosol and the nucleus [23], and ERP57 acts as a thiol oxidoreductase to catalyze the disulfide bond formations of the loaded glycoproteins [22]. And among this process, the glycosylation and disulphide bond status of the luminal domain of ATF6 can be utilized as novel sensing mechanisms for the activation of the UPR, and the cleavage to generate the active nuclear form of ATF6, and transported from ER to Golgi and then to the nucleus, to up-regulate the expression of GRP78 and ERP57 and reduce the unfolded protein load [24,25].

However, when ER homeostasis cannot be restored, the pro-apoptotic process is irreversibly induced [26], executed by ER resident Caspase 12 [27], which is a key signal involved in ER stress-induced apoptosis, localized on the cytoplasmic side of the ER, and Nakagawa et al. have been found that has been cells from Caspase 12 deficient mice are resistant to apoptosis triggered by the known ER stress agents [28]. Caspase 12 is specifically activated when ER stress is induced, and the activated Caspase 12 will enter cytoplasm from ER, gradually activate Caspase 9 and Caspase 3, and eventually induce ER stress-induced apoptosis. As during ER stress, activation of ATF6 is known to specifically up regulate chaperones, PDIs, as well as Caspase 12, and consequently lead to apoptosis [29], thus, the aim of this study was to investigate whether endoplasmic reticulum -related pathway is involved in SPS induces apoptosis in the mPFC of PTSD rats by examining the expression levels of ATF6 alpha (ATF6α), two important downstream molecular chaperone: GRP78 and ERP57, and apoptotic factors Caspase 12, Caspase 9, and Caspase 3.

## Methods

### Ethics statement

The study was approved by the ethics committee of China Medical University. All Wistar rats were maintained under clean grade. Housing and experimental protocols were in accordance with the Chinese Regulations for the Administration of Affairs Concerning Experimental Animals. All efforts were made to minimize the suffering of experiment animals during the procedures.

### Experimental animals

A total of 80 male Wistar rats, weighing 150-180 g, about 6 - 7 weeks old, were purchased from the Department of Laboratory Animals, China Medical University, and were pair-housed in a room with temperature of 22 ± 2°C and humidity of 55 ± 5%. All rats were housed under a reversed 12 h:12 h light/dark cycle (lights off at 10.00 a.m) and given standard food pellets and water.

### Groups and establishment of SPS model

After lab adaptation and handling, the rats were randomly assigned to one of four groups of twenty. One group served as a normal control (control), while others were SPS groups. The control group remained in their home cages with no handling until them were killed for test, and at the same time other rats were sacrificed at 1, 4 and 7 days after SPS, respectively called SPS1d group, SPS4d group and SPS7d group. The SPS model was created as described previously [30], which was described in “Advances in Basic and Clinical Research” international conference launched by Japanese Ministry of Education in 2005. Briefly, rats were restrained for 2 h and immediately forced to swim for 20 min in water (24°C). After a 15-min rest, they were anaesthetized by ether and then laid in their cages without disturbance until the experimental manipulations.

### Behavioral testing- Morris Water Maze (MWM) test

To measure learning and spatial memory performance, rats were tested in the Morris water maze (MWM) which based on the classic Morris protocol. Keep the water temperature (25 ± 1)°C, and surrounding environment was quiet and with constant light source. A 6-day testing study was performed as previously described in detail spatial reference and working memory deficits assessed in the water maze fixation and sections making [31]. Briefly, rats were initially brought into a quadrant (not containing the platform), facing the wall of the pool, to find the submerged platform within 120s. They were then allowed to stay on the platform for 20s. For learning performance, rats had four 120s learning trials daily each starting one of four quadrants in a random manner, with a 20s interval between trials. The escape latency to find the platform, calculated by averaging four trial values, was used to represent learning performance. Each rat was tested 6 consecutive days for learning performance. Spatial memory was evaluated at day 7 using the probe test, i.e., the platform was removed and animals were placed at a novel position. Average time spent in the target quadrant (platform removed) was calculated as an index for spatial memory.

### Brain tissue preparation and immunoflourescence assay for ATF6α

Five rats of each group were deep anesthetized with Nembutal (30 mg/kg, i.p.), and then infused with 200–300 ml of pre-cold saline through the ascending aorta, followed by 300 ml of 4% pre-cold paraformaldehyde (PFA) in 0.01 M PBS. The whole brain was rapidly removed and dissected on ice, followed by 6–10 h of post-fixation in 4% PFA at 4°C. Then the brains were embedded in paraffin. Samples were cut into 7 um thick and mounted on glass slides until to the morphological studies. Dewaxed sections were incubated with mouse polyclonal antibody against ATF6α (Santa Cruz, USA, 1:150) overnight at 4°C. After three times washing, the sections were incubated with CY3 anti-mouse IgG (Company of Zhongshan Goldenbridge, Beijing, China, 1:200) for 0.5 h at room temperature. After being washed in PBS and mounted. Confocal laser scanning microscope was applied for localization observation, and five slides were randomly selected from each group, each slide was randomly selected five visual fields in mPFC(×400), and eventually we recorded the optical density (OD) of positive cells in each field to evaluate the average of OD, andanalyzed using the MetaMorph/DPIO/BX41 morphology image analysis system.

### Immunohistochemical analysis for GRP78 and ERP57

Dewaxed sections were incubated with 0.3% hydrogen peroxide for 10 min, and later boiled in 10 mM citrate buffer (pH 6.0) in a water bath at 99°C for 5 min. Tissue sections were then incubated with the primary antibodies overnight at 4 8C. The antibodies were used at dilutions of 1:500 for GRP78 (Santa Cruz, USA) and ERP57 (Santa Cruz, USA). Then the sections were incubated with two-step IHC detection reagent (PV6001 and PV6002, Company of Zhongshan Goldenbridge, Beijing, China) at 37°C for 30 min. A brown color appeared in the slices after 3, 3′-diaminobenzidine colorization. Slices were then dehydrated and mounted with neutral gum. To assess nonspecific staining, a few sections in every experiment were incubated in PBS without primary antibody. Five slides were randomly selected from each group. Each slide was randomly selected five visual fields in mPFC (×400). We recorded the optical density (OD) of positive cells in each field to evaluate the average of OD, and analyzed using the MetaMorph/DPIO/BX41 morphology image analysis system.

### Western blotting to detect the protein expression of GRP78, ERP57

Five rats of each group were decapitated, and the whole brains were removed and immediately placed in an ice-cold dish. Then the mPFC was dissected according to the atlas [32] by use of a stereomicroscope and was quickly frozen in liquid nitrogen and stored-80°C. The mPFC was homogenized with a sample buffer containing 200 mM TBS, pH 7.5, 4% SDS, 20% glycerol, 10% 2-mercaptoethanol and denatured by boiling for 5 min. The protein fraction (50 μg/lane) prepared from each sample was separated by 8% (w/v) gradient sodium dodecyl sulfate(SDS)-polyacrylamide gel electrophoresis (PAGE) and electroblotted to a PVDF membrane (Millipore, Bedford, MA, USA) from the gel by a semi-dry blotting apparatus (Bio-Rad Laboratories, Inc., Hercules, CA, USA). After blocked with 5% dried skim milk, the membrane was incubated with I antibody (anti-GRP78, Santa Cruz, USA, 1:1000; anti-ERP57, Santa Cruz, USA, 1:1000; anti-GADPH, Zhongshan Glodenbridge Biotechnology, China; 1:1000) for 24 h at 4°C and II antibody ( Boster Biological Technology Ltd., 1:3000) for 2 h at room temperature. Blots were subjected to autoradiography (ECL reagents, Amersham Pharmacia Biotech, Buckinghamshire, UK). The optical density (OD) was analyzed on the Gel Image Analysis System. The expression of GRP78 and ERP57 were determined by calculating the OD ratio of GRP78/GADPH and ERP57/GADPH.

### Terminal deoxynucleotidyl transferase dUTP Nick-end labeling (TUNEL) staining

TUNEL staining was also carried out in order to further verify the mPFC apoptosis according to the kit instruction (KeyGEN BioTECH). Five slides were randomly selected from the control group and the SPS-7d group, and in each slide, five visual fields (×400) in the mPFC were randomly selected. The number of TUNEL-positive cells was counted and The TUNEL-positive cells rate was calculated to equal (the number of TUNEL − positive cells/total cells) × 100%.

### Total RNA extraction and RT-PCR

Tissues from three rats of each group were obtained as above and was quickly frozen in liquid nitrogen and stored -80°C. Total RNA was extracted from the frozen mPFC using Trizol (Invitrogen, USA) Kit according to the manufacturer’s instructions. 1 μg of total RNA was reverse transcribed into cDNA, and the cDNA concentration and purity were determined by measuring OD_260_ and OD_260/280_ ratio. Then cDNA was amplified using an RNA PCR Kit(AM Ver.3.0, TaKaRa bio, Otsu, Japan). All primers were designed using DNA star Primer Select program (Lasergene, Madison, WI, USA) and synthesized by Shanghai Sangong (Shanghai, China) according to the serial number from Genbank, and the sequence of each gene was shown in Table 1. The amplification profile of GRP78 and caspase 9 included: i) denaturation at 95°C for 4 min, ii) 30 additional cycles at 94°C for 45 sec and then 45 sec at 55°C, 72°C for 60 sec, and iii)extension at 72°C for 5 min. The amplification profile of ERP57 included: i) denatureion at 94°C for 4 min, ii) 30 additional cycles at 94°C for 45 sec and then 45 sec at 55°C, 72°C for 60 sec, and iii) extension at 72°C for 5 min. The amplification profile of caspase 12, and caspase 3 included: i) denatureion at 94°C for 4 min, ii) 35 additional cycles at 94°C for 45 sec and then 45 sec at 55°C, 72°C for 60 sec, and iii) extension at 72°C for 5 min. GAPDH mRNA used as an internal control was co-amplified with them. The PCR products were separated on 2.0% agarose gel by electrophoresis and the density of each band was analyzed on the Gel Image Analysis System (Tanon 2500R, Shanghai, China). The levels of these were normalized by GAPDH.

**Table 1:** **All primers for RT-PCR**

**Name**	**Primer**	**Product size**
GRP78	sense 5′ -CCAAGAGAGGGTTCTTGAATCTCG -3′	181 bp
	antisense 5′ -ATGGGCCAGCCTGGATATACAACA -3′	
ERP57	sense 5′ -GTGTTGGAACTGACGGACGA -3′	114 bp
	antisense 5′ -GGCAAGCCTCTTGCAATGTC -3′	
Caspase12	sense 5′ -GCACATTCCTGGTCTTTATGTCCC -3′	242 bp
	antisense 5′ -TTCCTCATCTGTATCAGCAGTGGC -3′	
Caspase9	sense 5′ -TGGTGGTGAGCAGTTTGACC -3′	189 bp
	antisense 5′ -CCTGGGAAGGTGGAGTAGGA -3′	
Caspase3	sense 5′ -CGGACCTGTGGACCTGAAAA-3′	219 bp
	antisense 5′ -TAGTAACCGGGTGCGTAGA -3′	
GAPDH	sense 5′ -ACTTTGGCATCGTGGAAGGG-3′	264 bp
	antisense 5′ -ACTTGGCAGGTTTCTCCAGG -3′	

### Statistical analysis

All data were analyzed by one-way analysis of variance (ANOVA) using the Tukey’s test to adjust for multiple comparisons or student’s t test where appropriate using SPSS 17.0 software. Data from multiple experiments were averaged and expressed as mean values ± SEM. A value of P < 0.05 was considered as statistically significant.

## Results

### Prolonged escape latency was observed in SPS rats from MWM Test

**Figure 1:**
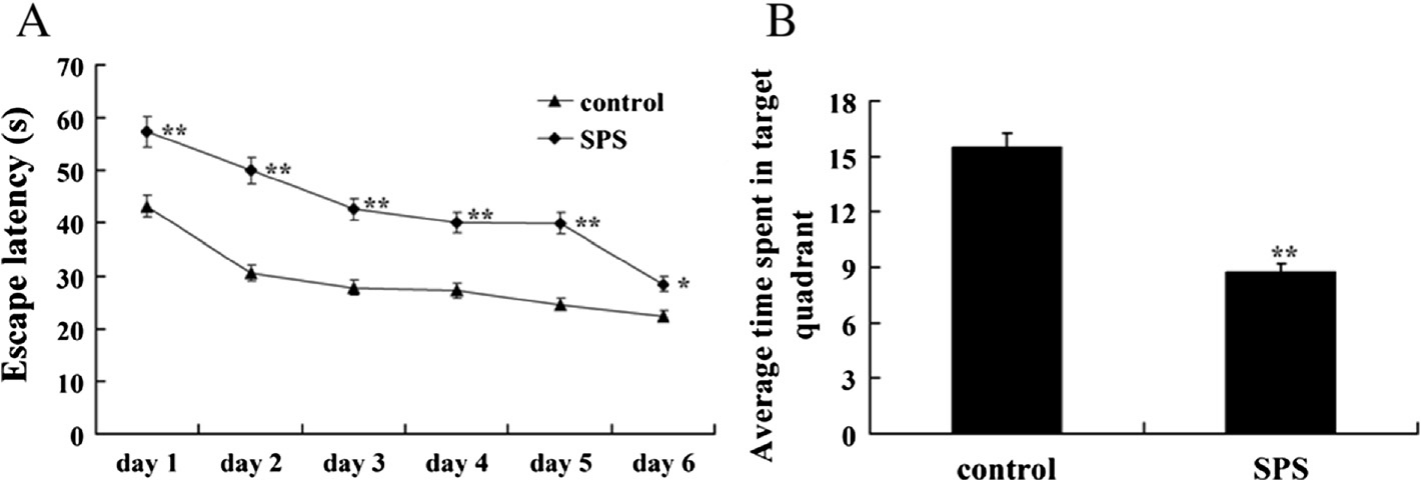
**Results of the MWM test. (A)**: Escape latency of three group rats in different test days. **(B)**: Average time spent in the target quadrants. n = 5 for each group, and statistical analysis was carried out by Tukey test. ^**^P < 0.01 compared to control group.

The results of MWM test are illustrated in Figure 1. Compared to control group, a striking increase of the escape latency was observed in SPS rats at day 1 through day 6 (Figure 1A). When the platform was removed from the pool at day 7 for spatial memory testing, a significant effect was observed across the two groups, the results showed that SPS rats had much less time spent in target quadrant compared to the control rats (Figure 1B), and revealed that SPS model was successfully copied, which had provided guarantee for subsequent studies.

### ATF6α was activated in response to SPS

**Figure 2:**
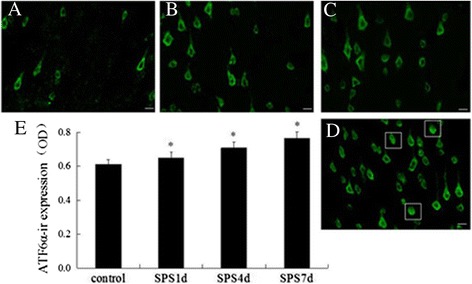
**Immunoflourescence observation of ATF6α visualized localization.** ATF6α-immunoreactivity (ir) in the mPFC of SPS rats in different groups (**A-D**, magnification × 400). **A**: the control group; **B**: the SPS1d group; **C**: the SPS4d group; **D**: the SPS7d group; **E**: the quantity of ATF6α-ir expression (OD). n = 5 for each group, scale bar = 30 um, and statistical analysis was carried out by Tukey test. ^*^P < 0.05 versus control.

ATF6 is an ER-membrane-bound transcription factor, a typical ER stress transducer and is localized to ER membranes [33]. It is activated by ER stress by cleavage from the membrane, moved to the nucleus and activated transcription [33,34]. In the current study, we hypothesized that SPS would result in the activation of ATF6α. To address this hypothesis, the effect of SPS on the expression of ATF6α was examined by immunoflourescence analysis. As shown on Figure 2A, ATF6α was present in the normal control rats and showed diffused cytoplasmic localization, whereas obvious increases of ATF6α were detected in response to SPS (Figure 2B-D and 2E), started at the early stage and persistently increased till 7 days. And all these results suggested that ATF6α was activated in response to SPS.

### Upregulation of GRP78 in the mPFC neurons after SPS exposure in immunohistochemical experiment

**Figure 3:**
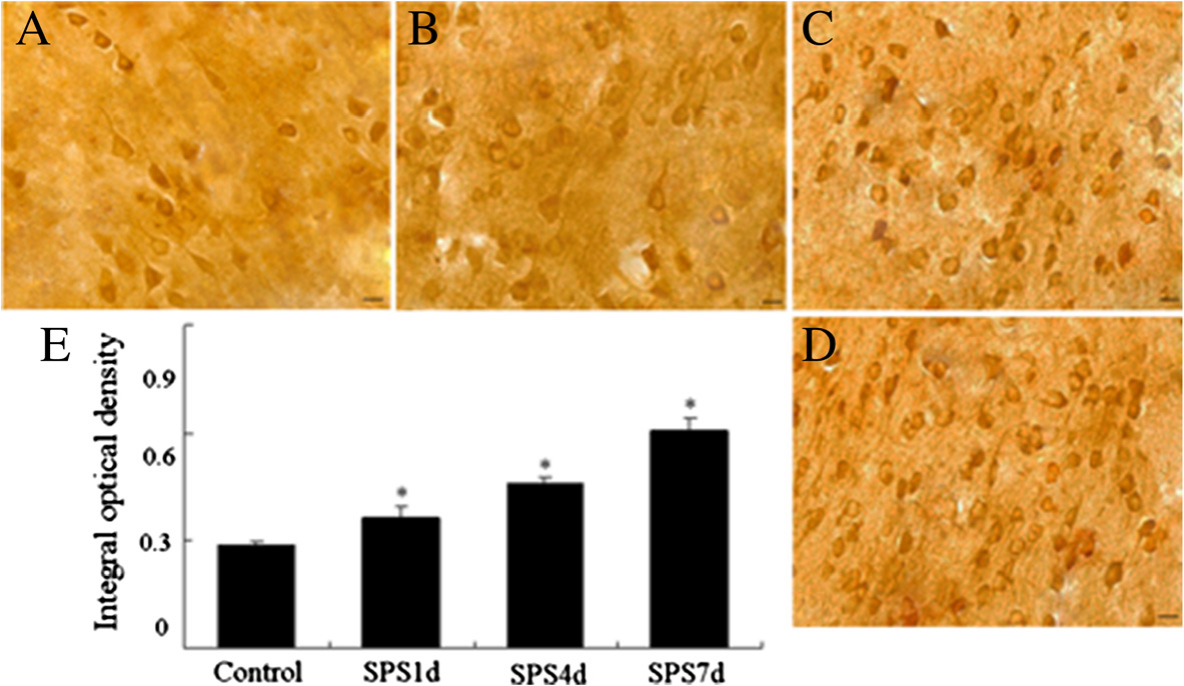
**Immunohistochemical observation of GRP78.** GRP78-immunoreactivity (ir) in the mPFC of SPS rats in different groups (**A-D**, magnification × 400). **A**: the control group; **B**: the SPS1d group; **C**: the SPS4d group; **D**: the SPS7d group; **E**: the quantity of GRP78-ir expression (OD). n = 5 for each group, scale bar = 30 um, and statistical analysis was carried out by Tukey test. ^*^P < 0.05 versus control.

GRP78 is a key regulator of ER stress transducer-ATF6α, and plays critical cytoprotective roles in neurodegenerative diseases [35,36]. To address the contribution of ATF6α activation, GRP78 was detected with immunohistochemistry assays in the current study. As shown in Figure 3A, GRP78 (ir) was distributed mainly in the cytoplasm of the mPFC neurons in the control group. All SPS groups showed higher immunoreactivity than the control groups (shown in Figure 3B-3D and 3E), and at SPS7d after SPS, the GRP78-ir positive cells reached a peak (Figure 3D and 3E), which revealing an upregulation of GRP78 in the mPFC neurons in response to SPS.

### Increase of ERP57 in the mPFC neurons after SPS exposure in immunohistochemical assay

**Figure 4:**
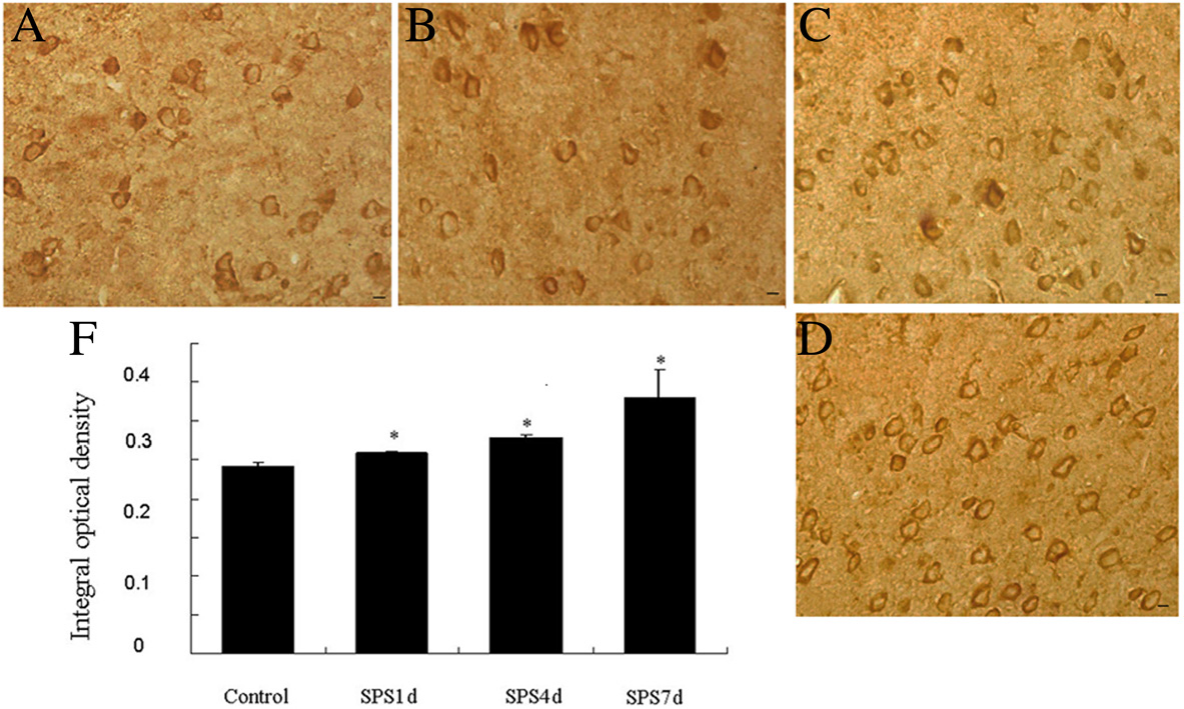
**Immunohistochemical observation of ERP57.** ERP57-immunoreactivity (ir) in the mPFC of SPS rats in different groups (**A-D**, magnification × 400). **A**: the control group; **B**: the SPS1d group; **C**: the SPS4d group; **D**: the SPS7d group; **E**: the quantity of ERP57-ir expression (OD). n = 5 for each group, scale bar = 30 um, and statistical analysis was carried out by Tukey test. ^*^P <0.05 versus control.

During ER stress, activation of ATF6α is also known to specifically up regulate PDIs, such as ERP57 [29], thus we determined to detect the protein expression of ERP57 after SPS exposure by immunohistochemistry assays. As 1 day after SPS, increased expression of ERP57 was observed from Figure 4B and 4E, then gradually climbing, and peaked at 7 days after SPS (Figure 4B-4D and 4E), indicative of an increase of ERP57 in the mPFC neurons in response to SPS.

### SPS triggered increases in the protein levels of GRP78 and ERP57

**Figure 5:**
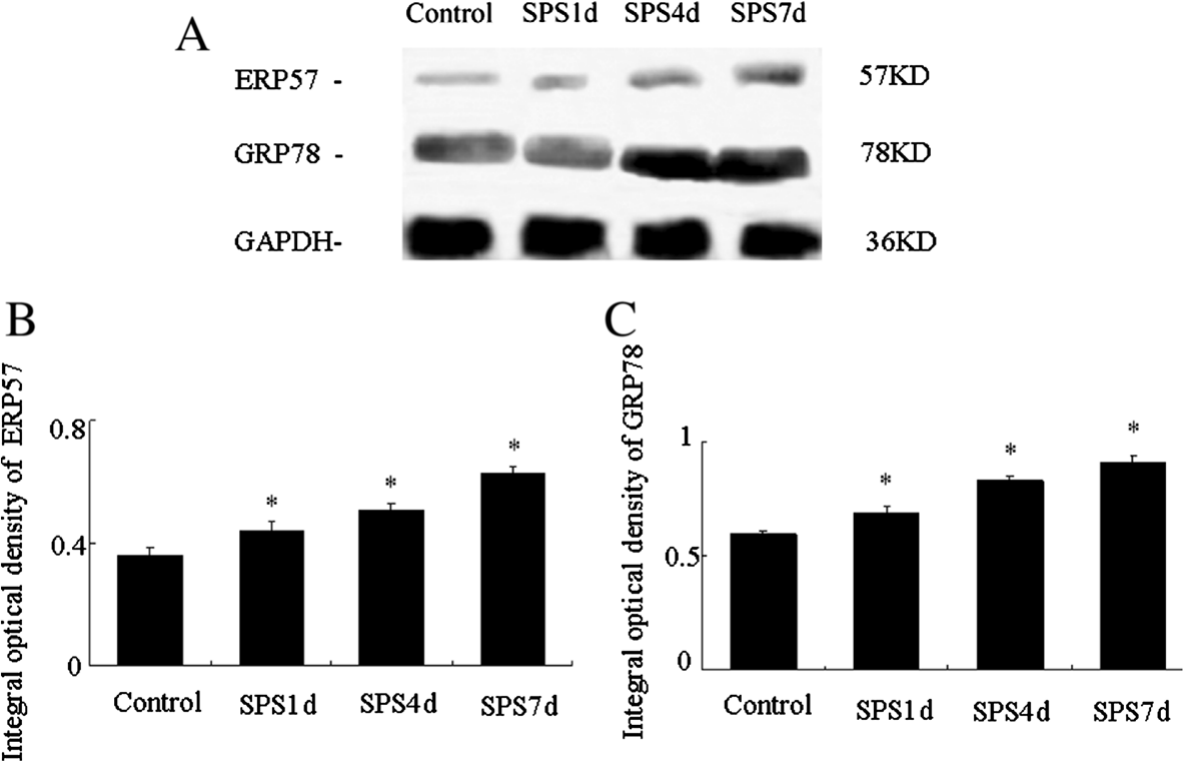
**Changes of GRP78 and ERP57 levels in the mPFC of SPS rats detected by Western blotting.** Typical Bands by Western blotting were shown in **(A)**, and relative analysis of GRP78 and ERP57 were shown in **(B)** and **(C)**, respectively. Statistical analysis was carried out by Tukey test. ^*^P <0.05 versus control.

Western blotting was also used to detect the protein expressions of GRP78 and ERP57 in mPFC of PTSD rats. Molecular weights of GRP78, ERP57 and GADPH were 78 kD, 57 kD and 36 kD, respectively, and the clear representative bands were shown in Figure 5A. The protein expression of GRP78 and ERP57 in the SPS groups showed a marked increase compared to that of the control group, and peaked at 7 days after SPS stimulation (Figure 5B and 5C). These results were consistent with them of immunohistochemical staining analysis, and suggested SPS triggered inductions of GRP78 and ERP57.

### SPS induced persistently upregulations in the transcripts levels of GRP78 and ERP57

**Figure 6:**
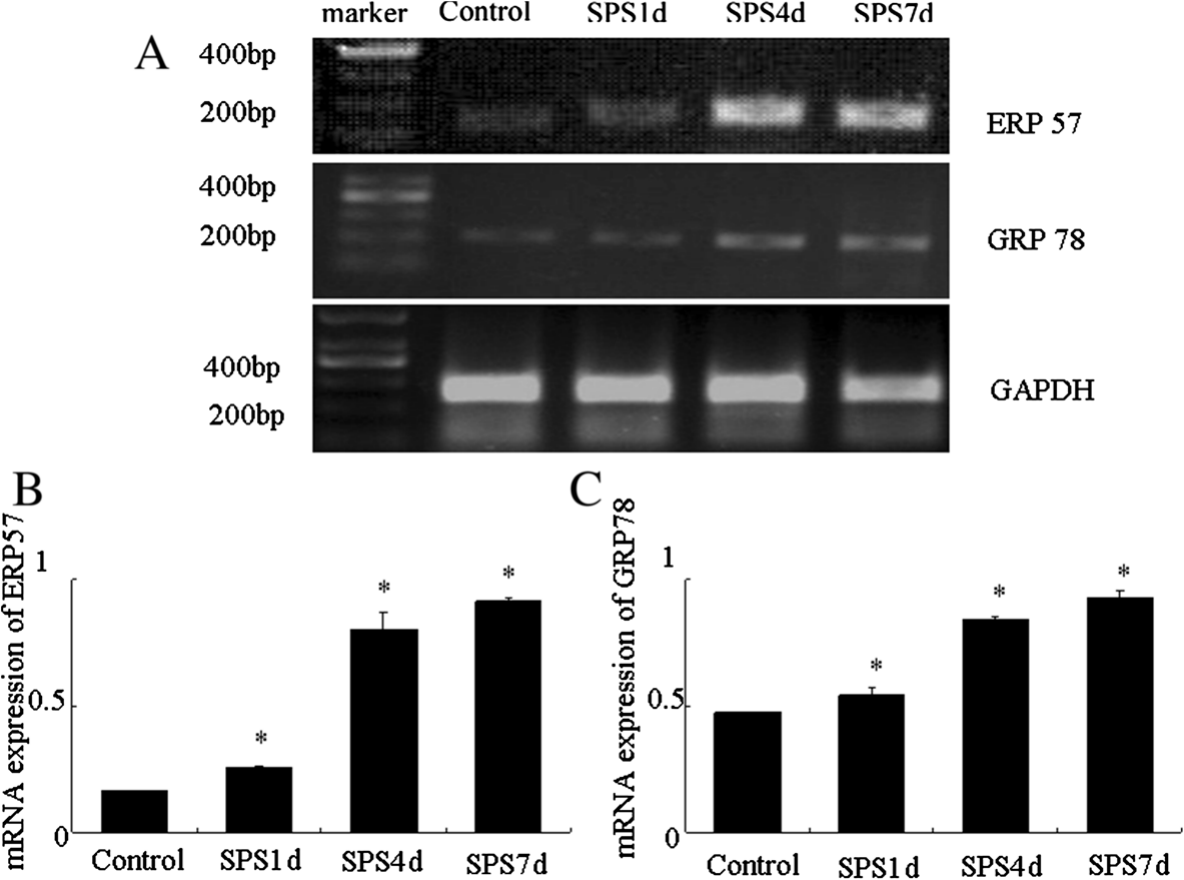
**Difference in GRP78 and ERP57 mRNA levels in control rats and SPS rats detected by RT-PCR.** Representative gel pattern of cDNA were shown in **(A)**, and relative amount of GRP78 and ERP57 mRNA were shown in **(B)** and **(C)**, respectively. Statistical analysis was carried out by Tukey test. ^*^P < 0.05 versus control.

Furthermore, we also examined an increase in total transcripts levels of GRP78 and ERP57 in the mPFC by RT-PCR. In control rats, very low level of GRP78 and ERP57 mRNA were detected (Figure 6A), however, the mRNA expression of GRP78 and ERP57 both had a persistently significant increase in SPS rats (Figure 6A), expecially at 4 days and 7 days after SPS (Figure 6B and 6C). These results revealed SPS induced persistently upregulations in the transcripts levels of GRP78 and ERP57.

### SPS induced cell death through ER stress and activation of caspases

**Figure 7:**
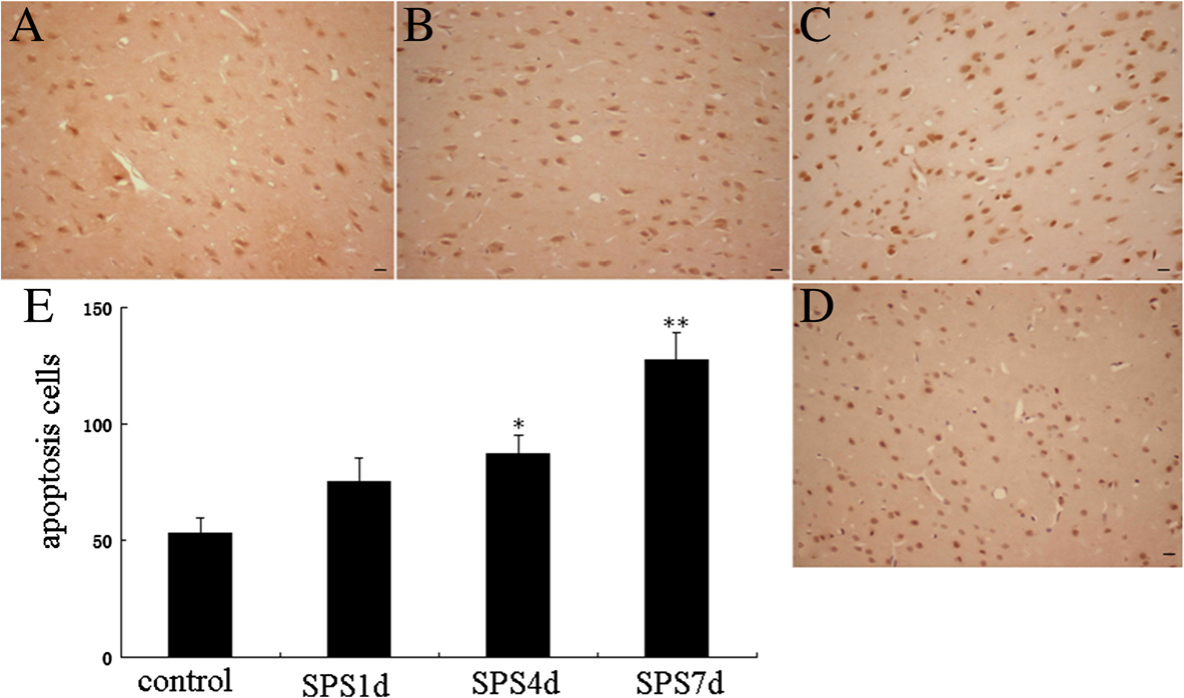
**Apoptosis detected by TUNEL assays.** The Representative images were shown in (**A-D**, magnification × 400), **A**: the control group; **B**: the SPS1d group; **C**: the SPS4d group; **D**: the SPS7d group; and **E** shows quantification of apoptosis cells. Statistical analysis was carried out by Tukey test. ^*^P < 0.05 versus control, ^**^P < 0.05 versus control.

**Figure 8:**
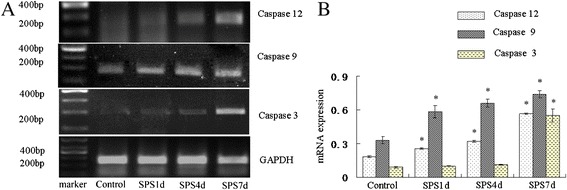
**RT-PCR of Caspase 12, Caspase 9 and Caspase 3 in the mPFC of SPS rats.** The Caspase 12, Caspase 9 and Caspase 3 mRNA expression **(A)** and the results from their quantitative analysis **(B)**. Statistical analysis was carried out by Tukey test. ^*^P < 0.05 versus control.

Just as shown as the Figure 7, brown particles presented in the nucleus under microscopy were apoptosis-positive cells detected by TUNEL staining. The control group showed a small amount of apoptosis-positive cells and has a lighter colour. However, 7 days after exposed to SPS, the amount of apoptosis-positive cells increased obviously and the color of brown particles became deeper, and there was a significant difference in the number of apoptosis-positive cells compared with that of the control group (shown in Figure 7E), which further suggested the existence of the mPFC apoptosis. And to see the changes in cellular agents related to ER stress and apoptosis, then tissue extracted from the mPFC were detected by RT-PCR, and the results showed that the mRNA level of the executor of ER apoptosis, Caspase 12, was persistently increased till 7 days after SPS (Figure 8A and 8B), which suggested Caspase 12 was activated by SPS, revealing signs of ER stress-induced apoptosis. Meanwhile, the mRNA amounts of Caspase 9 was gradually up regulated till 7 days after SPS exposure (shown in Figure 8B) at 7 days after SPS exposure, and the mRNA level of Caspase 3 had no significant difference compared to the control group at the early stage of SPS, however, there was dramatically increase at 7 days after SPS exposure (shown in Figure 8B). The up-regulation of the mRNA level of Caspase 9 and Caspase 3 suggested Caspase 9 and Caspase 3 were activated after SPS, and all those results indicate that ER stress and caspase activation contribute to SPS and SPS-induced apoptosis.

## Discussion

PTSD is a stress-related mental disorder that can develop in individuals who have been exposed to an event or events that involved the threat of death or serious injury and reacted with intense fear, helplessness or horror [1]. Although enormous progress has been made over the past decade in understanding on the molecular mechanism of PTSD, the exact pathogenesis remains obscured. Several magnetic resonance imaging (MRI) studies have reported decreased frontal cortex volume in PTSD [37-39] and decreased volume in medial prefrontal regions [40-43], some functional neuroimaging studies have typically reported less activation or even deactivation in ventral mPFC regions during traumatic script-driven imagery in PTSD [6-8]; and animal data also have demonstrated the vmPFC is critically involved in the extinction of the acquisition and expression of conditioned fear [44-46], particularly in the recall of extinction after a long delay [47,48]. These reports above have suggested that mPFC has been implicated in the pathogenesis of PTSD, and thus in this paper, we focused on this structure in order to understand further the pathological mechanism of PTSD.

SPS is a model for PSTD employed extensively [49,50], which determined at the International PTSD Scientific Meeting held by Japanese Ministry of Education in 2005. Animal experimental researches verify that rats after SPS exposure have dysfunction in hypothalamic-pituitary-adrena (HPA) axle, changes in ethology, activity habits, memory abilities and environmental adaptability reduces, in conformity with clinical manifestation of human PTSD. And SPS could also mimic the specific neuroendocrinological abnormalities observed in PTSD patients, by conducting in three stages: restraint for 2 h, forced swim for 20 min, and ether anesthesia, which correspond to psychological, physiological, and endocrinological stress, respectively. In current experiment, we set up SPS model and carried out MWM test to confirm whether the model succeeded. From Figure 1A and B, we could observed that the escape latency was prolonged in SPS rats at day 1 through day 6, and when the platform was removed from the pool at day 7 for spatial memory testing, SPS rats had much less time spent in target quadrant compared to the control rats. All these results revealed that SPS model in this study was successfully copied, which had provided guarantee for subsequent studies.

Given in our previous studies, our research teammate Wen et al. [51] have proved that there were abnormal expression of Ca^2+^-CaM-CaMKIIα pathway in the mPFC neurons, and dysfunction of cytosolic Ca2+ concentration was taken as one of inducement of ER stress, we deduced that ER stress would be induced by dysfunction of Ca^2+^-CaM-CaMKIIα pathway in the mPFC neurons. And at the same time, our teammate Yana et al. have found that there were expression changes of bcl-2 and bax in the mPFC neurons of SPS-model rats [10]; Zhao et al. [52] also have examined the apoptosis morphological changes by transmission electron microscopy (TEM) and demonstrated the existence of apoptosis in the mPFC neurons of rats after SPS. As ER stress would induce apoptosis, thus in this paper we hypothesized that ER stress and ER-related apoptosis would be involved in the molecular mechanisms of SPS-induced apoptosis in the mPFC neurons of PTSD-like rats.

And as we all know, ATF6α is a typical ER stress transducer [53,54], and to elucidate its potential alterations in the mPFC neurons after SPS exposure, firstly we evaluated the levels of ATF6α by the use of immunofluorescence assay. As shown in Figure 2B-D and 2E, obvious increases of ATF6α were detected in response to SPS, started at the early stage and persistently increased till 7 days, which indicated that SPS activated ER stress transducer ATF6α. And as at the beginning of ER stress ATF6α pathway triggers and results in the upregulation of ER-derived chaperones to rescue cells from ER stress [18,24,25], next we detected the expression of a key regulator of ER stress GRP78 by immunohistochemistry, western blotting and RT-PCR. Our results showed that the protein expression of GRP78 increased 1, 4 days, peaked on 7 days after SPS stimulation, and the mRNA expression changes of GRP78 were similar to the trend of its protein changes, which revealed that there was accumulation of GRP78 in the mPFC neurons in response to SPS. The accumulation of GRP78 are beneficial because on one hand, as a chaperone, GRP78 recognizes and binds to the proteins with hydrophobic residues in the unfolded regions to assist with the correct folding of the unfolded proteins [55]; and on the other hand, GRP78 also can form in a large multi-protein complex with a set of ER molecular chaperones, GRP94, PDI, ERp57, and so on, which forms an ER chaperoning network processing the unfolded protein substrates [56].

Given the ATF6α pathway also can trigger and result in the upregulation of protein-folding enzymes ERP57 [24,25,29], we also observed the protein and mRNA expression of ERP57, and our results showed that there were increased expressions in the protein level and the mRNA level of ERP57. ERP57 is a 58-kDa protein with significant homology to protein disulfide isomerase and has two thioredoxin-like domains and is suspected to function as thiol-dependent oxidoreductase [57]. ERP57 can interact with glycoproteins such as calnexin and calreticulin, playing an important role as a molecular chaperone during glycoprotein biosynthesis and folding [22], and also can promote the formation of intra- or intermolecular disulfide bonds during glycoprotein folding [58-60] to cope with excessive protein folding load and re-establish cellular homeostasis, and it has reported that ERP57 has shown clinical applications to endoplasmic reticulum stress associated diseases and cancer [61], disruption of ERP57 in mouse is lethal. And during ER stress, when the ATF6α pathway is activated, the active nuclear form of ATF6α is transported in the nuclear and then induces the upregulation of ERP57 to copy with the ER stress, which just as we saw from our results that the expression of ERP57 was up-regulated in the neurons of the mPFC in response to SPS, and comprehensive the above results of GRP78, we can draw a conclusion that SPS triggered not only the induction of GRP78, but also the induction of ERP57, which both are the downstream of the ATF6α pathway and indicators of ER stress, and that is, ATF6α pathway ER stress was induced in the mPFC neurons in response to SPS.

As when ER stress is prolonged or severe, apoptotic pathways can be activated [62,63], next we evaluated apoptosis using the TUNEL assay and performed RT-PCR analysis of the total transcripts levels of Caspase 12, which is a key signal involved in ER stress-induced apoptosis [28,29], and we observed the number of apoptosis-positive cells increased obviously 7 days after exposed to SPS (shown in Figure 7), and the mRNA of Caspase 12 also was persistently increased till 7 days after SPS, revealing signs of ER stress-induced apoptosis. And furthermore, as the activated Caspase 12 will enter cytoplasm from ER, and gradually activate Caspase 9 and Caspase 3, then we also observed the mRNA expression changes of Caspase 9 and Caspase 3, and found that there were dramatically increase in the mRNA level of them at 7 days after SPS exposure (shown in Figure 8B), which suggested Caspase 9 and Caspase 3 were also activated after SPS. Taken together, we could draw a conclusion that SPS induced cell death through ER stress and activation of caspases, ER stress-induced apoptosis contributed to SPS. However, as the activation of Caspase 3 and 9 also can occur by mitochondrial apoptotic pathway, therefore, it is necessary to identify whether the mitochondrial apoptotic pathway also play key roles in inducing apotosis of the mPFC after SPS, which is one of the limitations in this experiment. And furthermore, it is still required to elucidate the precise mechanism underlying SPS-induced ER stress and ER stress-related apoptosis.

## Conclusion

In summary, our studies demonstrated that SPS triggered the activation of ATF6α and the increase of GRP78 and ERP57, and also up regulated the transcripts levels of Caspase 12, Caspase 9 and Caspase 3. Our results indicated that SPS induced ATF6α-dependent ER stress and ER stress-related apoptosis in the mPFC neurons, which indicated that the endoplasmic reticulum pathway may be involved in PTSD-induced apoptosis and may provide important information for the pathogenesis and treatment of PTSD.

## Abbreviations

ANOVA: Analysis of variance
con: Control group
DAB: 3,30-diaminobenzidine
ER: Endoplasmic reticulum
GAPDH: Glyceraldehyde-3-phosphate dehydrogenase
GRP78: Glucose-regulated protein 78
IR: Immunoreactivity
OD: Optical density
PFA: Pre-cold paraformaldehyde
PTSD: Post-traumatic stress disorder
PVDF: Polyvinylidene fluoride
RT-PCR: Reverse transcription-polymerase chain reaction
SPS: Single-prolongedstress
UPR: Unfold protein response
